# Prognostic model development for risk of curve progression in adolescent idiopathic scoliosis: a prospective cohort study of 127 patients

**DOI:** 10.2340/17453674.2024.41911

**Published:** 2024-09-13

**Authors:** Marlene DUFVENBERG, Anastasios CHARALAMPIDIS, Elias DIARBAKERLI, Birgitta ÖBERG, Hans TROPP, Anna Aspberg AHL, Daphne WEZENBERG, Henrik HEDEVIK, Hans MÖLLER, Paul GERDHEM, Allan ABBOTT, CONTRAIS Study Group

**Affiliations:** 1 Department of Health, Medicine and Caring Sciences, Unit of Physiotherapy, Linköping University, Linköping; 2 Department of Clinical Science, Intervention and Technology (CLINTEC), Division of Orthopaedics and Biotechnology, Karolinska Institutet, Stockholm; 3 Department of Reconstructive Orthopaedics, Karolinska University Hospital Huddinge, Stockholm; 4 Department of Biomedical and Clinical Sciences, Linköping University, Linköping; 5 Center for Medical Image Science and Visualization, Linköping University, Linköping; 6 Department of Orthopaedics, Linköping University Hospital, Linköping; 7 Department of Orthopaedics, Ryhov County Hospital, Jönköping; 8 Stockholm Center for Spine Surgery, Stockholm; 9 Department of Orthopaedics and Hand Surgery, Uppsala University Hospital, Uppsala; 10 Department of Surgical Sciences, Uppsala University, Uppsala, Sweden

## Abstract

**Background and purpose:**

The study’s purpose was to develop and internally validate a prognostic survival model exploring baseline variables for adolescent idiopathic scoliosis curve progression.

**Methods:**

A longitudinal prognostic cohort analysis was performed on trial data (n = 135) including girls and boys, Cobb angle 25–40°, aged 9–17 years, remaining growth > 1 year, and previously untreated. Prognostic outcome was defined as curve progression of Cobb angle of > 6° prior to skeletal maturity. 34 candidate prognostic variables were tested. Time-to-event was measured with 6-month intervals. Cox proportional hazards regression survival model (CoxPH) was used for model development and validation in comparison with machine learning models (66.6/33.3 train/test data set). The models were adjusted for treatment exposure.

**Results:**

The final primary prognostic model included 127 patients, predicting progress with acceptable discriminative ability (concordance = 0.79, 95% confidence interval [CI] 0.72–0.86). Significant prognostic risk factors were Risser stage of 0 (HR 4.6, CI 2.1–10.1, P < 0.001), larger major curve Cobb angle (HR_standardized_ 1.5, CI 1.1–2.0, P = 0.005), and higher score on patient-reported pictorial Spinal Appearance Questionnaire (pSAQ) (HR_standardized_ 1.4, CI 1.0–1.9, P = 0.04). Treatment exposure, entered as a covariate adjustment, contributed significantly to the final model (HR 3.1, CI 1.5–6.0, P = 0.001). Sensitivity analysis displayed that CoxPH maintained acceptable discriminative ability (AUC 0.79, CI 0.65–0.93) in comparison with machine learning algorithms.

**Conclusion:**

The prognostic model (Risser stage, Cobb angle, pSAQ, and menarche) predicted curve progression of > 6° Cobb angle with acceptable discriminative ability. Adding patient report of the pSAQ may be of clinical importance for the prognosis of curve progression.

Adolescent idiopathic scoliosis (AIS) is a complex 3D-pathology of the spine and trunk with a Cobb angle of ≥ 10° in the frontal plane occurring during the time of puberty when there is rapid growth.

Despite the development of recommendations regarding thresholds for treatment indications [1], approximately 50% of patients will not have progress of scoliosis despite not receiving treatment [2,3]. This implies a need to improve prognostic models for identifying those at high risk of progression compared with those with non-progressive scoliosis to avoid under- and overtreatment.

Considering the etiology, a wide range of potential prognostic risk factors are summarized in the literature with inconsistent findings including; genetics and epigenetics, magnitude of the curve, curve type, curve flexibility, bone mineral density, disc wedging, age at diagnosis, Risser stage, open triradiate cartilage, Sanders skeletal maturity stages, premenarchal and maturity stages, and peak body height [4-7]. Existing prognostic models use different methods and definition of curve progression thresholds and are not conclusive for predicting long-term outcome of AIS [4,8,9]. Previous systematic review on predictors applying pooled prognostic characteristics for curvature progression in AIS indicated limited clinical application and low level of evidence [9]. A recent review concluded that the most relevant predictive factors were curve magnitude, skeletal maturity, and curve location [7].

To our knowledge, no previous study has covered patient characteristics, clinical measurements, and patient-reported measures of health-related quality of life aspects as risk factors at baseline. The potential for improved multivariable prognostic models for curve progression in AIS is crucial to support selection of treatment decisions regarding candidates for early curve management.

Our study aims to develop and internally validate a multivariable prognostic survival model exploring baseline factors for future risk of curve progression of more than 6° in adolescents’ idiopathic scoliosis with a Cobb angle of 25–40°.

## Methods

### Study design and participants

This study follows the TRIPOD guidelines (Transparent Reporting of a Multivariate Prediction Rule for Individual Prognosis or Diagnosis) [10].

This was a longitudinal prognostic cohort analysis within Conservative Treatment for Adolescent Idiopathic Scoliosis (CONTRAIS) study, which is a prospective multicenter randomized controlled trial (RCT) comparing 3 conservative treatment groups and has previously been described [2,11]. From January 2013 through October 2018, adolescents with idiopathic scoliosis were screened. Patients were continuously included during the study period.

Data from the CONTRAIS study [11] was utilized for prognostic model development with 135 enrolled patients treated at 6 study centers in Sweden. Inclusion criteria were girls and boys 9–17 years of age with idiopathic scoliosis, 25–40° Cobb angle of the major curve with apex at or caudal to T7 [12], not more than 1 year after menarche and skeletally immature with estimated remaining growth of at least 1 year [13]. Exclusion criteria were previous treatment for scoliosis, or inability to understand Swedish. Patients who were eligible but declined to participate in the study were offered standard care with a corrective thoracolumbar sacral orthosis (TLSO) to be worn at least 20 hours/day until skeletal maturity was confirmed.

After inclusion in the CONTRAIS study, consecutive block randomization (1:1:1) was performed into 1 of 3 groups with treatment prescription of: (i) 1 hour adequate levels of physical activity alone as control (PA) or 1 hour of physical activity combined with either (ii) Boston scoliosis night brace, night-time brace wear of 8 hours (NB) or (iii) a daily 30 minutes of scoliosis-specific exercise (SSE) that was accounted for in the total hour (see Supplementary data). All groups were followed up with the same outcome measures every 6 months until curve progression of 6° or less at skeletal maturity (success), or more than 6° before skeletal maturity (failure) [2,11].

### Factors in the prognostic survival model

Prognostic outcome. The dependent binary factor was defined as curve progression of 6° or less at skeletal maturity “no progress” (success) or curve progression of more than 6° as the event “progress” (failure) seen on 2 consecutive posteroanterior standing radiographs compared with the inclusion radiograph. This was not only to account for Cobb angle measurement error, but also the consequences of progression as a threshold for the need to transition to full-time bracing, which is a demanding treatment for patients with AIS. Radiographic data on Cobb angle was collected at baseline and at each 6-month follow-up. Skeletal maturity was defined as less than 1 cm growth of body height in 6 months. If progress was suspected, radiographs were assessed by 2 experienced investigators blinded to the treatment prior to any decision on progress. Radiographic measurements were conducted using bi-planar radiographs using the PACS clinical imaging tool (Sectra PACS, version 23.1, Linköping, Sweden). For patients in the NB group, the brace was not worn the night before the radiograph was conducted. The mean value of the 2 assessments on Cobb angle measurements of the largest curve was reported [2]. Patients who experienced progress were, instead of the assigned treatment, given the option to transition to standard care with a corrective TLSO.

**Figure 1:**
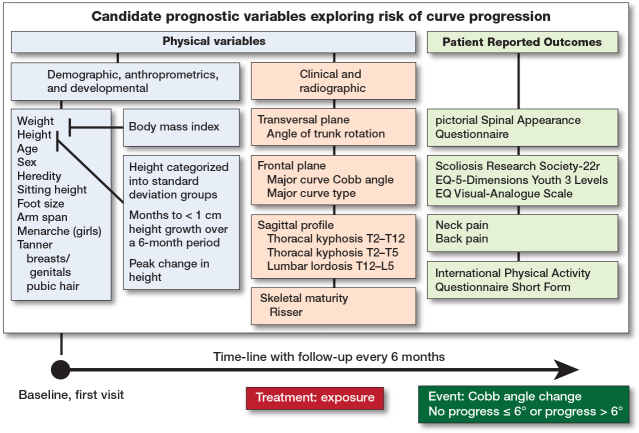
Candidate independent prognostic variables were measured at baseline. Height and weight were used to calculate body mass index. Height and age were used to calculate 3 normalized standardized height variables. Event was no progress if Cobb angle was ≤ 6° on reaching skeletal maturity or progress if curve progression > 6° occurred prior to skeletal maturity. Skeletal maturity was reached at less than 1 cm of growth in body height over 6 months. Time-to-event was measured for each patient from baseline at the first visit at pre-specified intervals of 6 months until event. Adjustment for treatment exposure was Boston scoliosis night brace versus Scoliosis-specific exercise and Control with adequate self-mediated physical activity alone.

Time-to-event was measured for each patient from baseline at the first visit (t0) until event (tx), at pre-specified intervals of 6 months (Figure 1).

**Figure 2:**
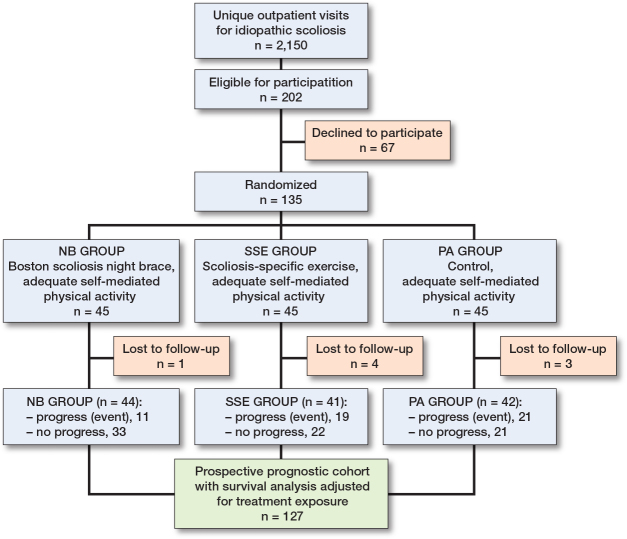
Enrollment of participants into the prognostic cohort survival analysis conducted within a randomized control trial (CONTRAIS) comparing 3 conservative treatments. From January 2013 through October 2018, 2,150 adolescents with idiopathic scoliosis were screened.

Independent variables and treatment exposure. The model was developed using the CONTRAIS data, from baseline at first visit, including 34 candidate prognostic independent variables described in detail [2,11] (Figure 2, Tables 1–3):

**Table 1:** Description of sample characteristics (N = 127) and candidate prognostic variables for the prognostic model development process

Prognostic variables at baseline	Type of variable	Values or categories	Mean (SD) or n (%)	Prognostic model development process			
				Step 1		Step 2	Step 3
				HR (CI)	P value		
Demographics, anthropometrics, and developmental measures							
Age, years	Continuous		13 (1.4) **^a^**	0.83 (0.64–1.07) **^b^**	0.2		
	Ordinal	9–10 (Ref.)	14 (11)		0.04	Yes	No
		11–12	60 (47)	0.99 (0.46–2.15)			
		13–15	53 (42)	0.44 (0.18–1.06)			
Sex	Binary	Girl; Boy (Ref.)	106 (83); 21 (17)	0.81 (0.41–1.63)	0.6		
Body mass index	Continuous		18.3 (2.7) **^a^**	0.87 (0.65–1.16) **^b^**	0.3		
	Ordinal	< 18.5 underweight (Ref.)	76 (60)		0.3		
		18.5–24.9 normal weight	47 (37)	0.65 (0.35–1.21)			
		25.0–29.9 overweight	4 (3)	0.41 (0.06–3.04)			
Heredity	Binary	Yes; No (Ref.)	70 (55); 57 (45)	0.84 (0.48–1.46)	0.5		
Height, cm	Continuous		158 (9.0) **^a^**	0.92 (0.71–1.20) **^b^**	0.5		
Sitting height, cm	Continuous		82 (5.1) **^a^**	0.76 (0.58–1.00) **^b^**	0.049		
Foot size, mean of left/right, cm	Continuous		24 (1.6) **^a^**	1.09 (0.83–1.43) **^b^**	0.5		
Arm span, cm	Continuous		160 (10.6) **^a^**	0.92 (0.71–1.20) **^b^**	0.5		
Menarche (boys coded as no)	Binary	No; Yes (Ref.)	81 (64); 46 (36)	3.06 (1.43–6.55)	0.004	Yes	Yes
Tanner scale, breasts/genitals	Ordinal	Stage I (Ref.)	7 (6)		0.1		
		Stage II	24 (19)	1.53 (0.53–4.40)			
		Stage III	67 (53)	1.10 (0.39–3.05)			
		Stage IV	26 (20)	0.29 (0.07–1.27)			
		Stage V	3 (2)	1.30 (0.15–11.7)			
	Binary	Stage I–II; III–V (Ref.)	31 (24); 96 (76)	1.56 (0.86–2.84)	0.1		
Tanner scale, pubic hair	Ordinal	Stage I (Ref.)	16 (13)		0.06		
		Stage II	22 (17)	1.41 (0.64–3.11)			
		Stage III	34 (27)	0.67 (0.29–1.56)			
		Stage IV	49 (39)	0.49 (0.21–1.14)			
		Stage V	6 (5)	0.32 (0.04–2.50)			
	Binary	Stage I–II; III–V (Ref.)	38 (30); 89 (70)	2.21 (1.26–3.90)	0.006	Yes	No
Normalized variables based on the Swedish population longitudinal reference values for height and age							
Height, cm, categorized	Ordinal	−5 SD to −1 SD (Ref.)	32 (25)		0.02	No	
into SD groups		0 SD	54 (43)	2.72 (1.27–5.83)			
		+1 SD to +5 SD	41 (32)	1.50 (0.63–3.57)			
	Binary	0 SD; Other (Ref.)	54 (43); 73 (57)	2.17 (1.24–3.79)	0.007	Yes **^d^**	No
Months to < 1 cm height growth over a 6-month period	Continuous		22 (16)	1.24 (0.97–1.59)	0.08	Yes **^c^**	No
Peak change in height	Binary	Before peak; At or after peak (Ref.)	28 (22); 99 (78)	1.20 (0.64–2.24)	0.6		

a Values are mean (SD).

b Continuous variables were standardized in Cox proportional hazards regression models.

*Step 1.* Results of univariate Cox proportional hazards regression models, where significant and clinical important variables (**^c^**) are selected into step 2.

*Step 2.* Most parsimonious variables (**^d^**) are selected for step 3, after checking for multicollinearity issues.

*Step 3.* A multivariable Cox proportional hazards regression model is developed using a backward conditional selection procedure.

SD: standard deviation; HR: hazard ratio; CI: 95% confidence interval; Ref.: reference category.

**Table 2:** Description of sample characteristics (N = 127) and candidate prognostic variables for the prognostic model development process

Prognostic variables at baseline	Type of variable	Values or categories	Mean (SD) or n (%)	Prognostic model development process			
				Step 1		Step 2	Step 3
				HR (CI)	P value		
Clinical and radiologic measures							
Angle of trunk rotation (°)	Continuous		11 (2.8) **^a^**	1.21 (0.91–1.61) **^b^**	0.2		
Major curve Cobb angle (°)	Continuous		31 (4.3) **^a^**	1.31 (1.00–1.71) **^b^**	0.048	Yes	Yes
Major curve type	Categorical	Thoracal (Ref.)	84 (66)		0.98		
		Thoracolumbar	16 (13)	0.92 (0.36–2.36)			
		Lumbar	27 (2)	0.87 (0.43–1.75)			
Sagittal profile (°)	Continuous						
Thoracal kyphosis T2–T12, n = 110			26 (14) **^a^**	1.05 (0.78–1.43) **^b^**	0.7		
Thoracal kyphosis T5–T12, n = 110			14 (11) **^a^**	0.84 (0.63–1.12) **^b^**	0.2		
Lumbar lordosis T12–S1, n = 113			−49 (12) **^a^**	0.95 (0.72–1.25) **^b^**	0.7		
Risser, skeletal maturity assessment of ossification of iliac apophysis (0–5) **^e^**	Ordinal	Stage 0 (Ref.)	67 (53)		< 0.001	No	
		Stage 1–2	26 (20)	0.20 (0.07–0.57)			
		Stage 3–4	34 (27)	0.22 (0.09–0.56)			
	Binary	Stage 0; 1–4 (Ref.)	67 (53); 60 (47)	4.73 (2.29–9.78)	< 0.001	Yes **^c^**	Yes
Treatment exposure, covariate adjusted for in the model							
Treatment exposure	Categorical	NB (Ref)	44 (35)		0.03	No	
		SSE	41 (32)	2.38 (1.13–5.02)			
		PA	42 (33)	2.62 (1.26–5.44)			
	Binary	SSE+PA; NB (Ref.)	83 (65); 44 (35)	2.50 (1.28–4.89)	0.007	Yes	Yes
Prognostic outcome, event							
Assessed according to major curve	Binary	Progress; No	51 (40); 76 (60)				
Cobb angle (°) change		progress (Ref.)					
Time-to-event	Continuous		20 (12) **^a^**				

e Zero patients with Risser stage 5.

For abbreviations, see Table 1. NB: Boston scoliosis night brace; SSE: scoliosis-specific exercise; PA: control with adequate self-mediated physical activity alone.

**Table 3:** Description of sample characteristics (N = 127) and candidate prognostic variables for the prognostic model development process

Prognostic variables at baseline	Type of variable	Values or categories	Mean (SD) or n (%)	Prognostic model development process			
				Step 1		Step 2	Step 3
				HR (CI)	P value		
Patient-reported outcomes on health-related quality of life aspects							
pSAQ (7–35)	Continuous		12 (3.1) **^a^**	1.33 (1.02–1.73) **^b^**	0.04	Yes	Yes
SRS-22r (1–5)	Continuous						
Function			4.8 (0.3) **^a^**	0.92 (0.70–1.20) **^b^**	0.5		
Pain			4.7 (0.6) **^a^**	1.09 (0.81–1.45) **^b^**	0.6		
Self-image			4.2 (0.6) **^a^**	0.93 (0.72–1.20) **^b^**	0.6		
Mental health			4.3 (0.6) **^a^**	1.10 (0.82–1.49) **^b^**	0.5		
EQ-5D-Y-3L index (−0.594 to 1)	Continuous		0.89 (0.18) **^a^**	1.06 (0.81–1.39) **^b^**	0.7		
EQ-VAS (0–100)	Continuous		88 (11) **^a^**	0.87 (0.67–1.12) **^b^**	0.3		
Back pain	Binary	Yes; No (Ref.)	42 (33); 85 (67)	1.03 (0.58–1.85)	0.9		
Neck pain	Binary	Yes; No (Ref.)	18 (14); 109 (86)	1.21 (0.57–2.57)	0.6		
IPAQ-SF (MET-min/week), n = 121	Continuous		2,666 (2,628) **^a^**	0.84 (0.60–1.17) **^b^**	0.3		

For abbreviations, see Table 1. pSAQ: pictorial part of Spinal Appearance Questionnaire; SRS-22r: Scoliosis Research Society-22 revised; EQ-5D-Y-3L: EQ-5 Dimensions Youth 3 levels index; EQ-VAS: EQ Visual Analogue Scale; IPAQ-SF MET-min/week: International Physical Activity Questionnaire Short Form Metabolic Equivalent of Tasks in minutes per week.

- Demographics, anthropometrics, and developmental, 14 variables: age (total group, and 9–10 vs 11–12 vs 13–15 years); sex (girls/boys); heredity (yes/no); body mass index (total group, and < 18.5 vs 18.5–24.9 vs 25.0–29.9); sitting height (cm); foot size, left/right (cm); and arm span (cm); and premenarchal (yes/no); Tanner, maturity stages for breast/genitals and pubic hair (stage 1, 2, 3, 4, 5, and 1–2 vs 3–5) (Table 1).
- Normalized measures from height and age, 4 variables: based on the Swedish population-based longitudinal reference values from birth to 18 years of age for height [13] the variables were constructed into “height” (cm) categorized into below, at, or above normalized standard deviation (SD) groups (−5SD to −1SD vs 0SD vs +1SD to +5SD and 0SD yes/no); “months to less than 1 cm height growth over 6 months” (expected number of months to maturity); and “peak change in height” (before peak vs at or after peak) (Table 1).
- Clinical and radiologic, 6 variables: angle of trunk rotation assessed with scoliometer (degrees); major curve Cobb angle; major curve type (thoracal, thoracolumbar, or lumbar); sagittal profile (thoracal kyphosis T2–T12 vs thoracal kyphosis T5–T12 vs lumbar lordosis T12–S1), and ossification of the iliac apophysis according to American Risser stages (stage 0 vs 1–2 vs 3–5, and 0 vs 1–5) (Table 2).
- Patient-reported outcomes on health-related quality of life aspects, 10 variables: perception of spinal appearance with the pictorial part of the Spinal Appearance Questionnaire (pSAQ) (7–35) [14]; Scoliosis Research Society-22 revised (SRS-22r) (1–5) [15], EQ-5-Dimensions-Youth-3L (EQ-5D-Y-3L) value set (index) [16], and EQ Visual-Analogue-Scale (EQ-VAS) (0–100) [17]; pain in back and/or neck (yes/no); and International Physical Activity Questionnaire Short Form (IPAQ-SF) in metabolic equivalent (MET-minutes/week) [18] (Table 3).

Covariate adjustment was based on a binary variable for treatment groups, NB vs SSE and PA, because NB prevented curve progression while the others did not [2] (Table 2).

### Statistics: model development and validation

The following criteria (step 1–3) were used during development of the primary model (Table 1–3):

*Step 1.* Using Cox proportional hazards survival model (CoxPH) on each candidate prognostic variable. All assumptions of proportional hazards were met in all univariate analyses. Expectation–maximization–imputation was used for missing data. We selected variables based on significant association with the prognostic outcome (P < 0.1). In addition, the 2 variables with normalized values on height were selected in accordance with clinical practice.

*Step 2.* Variables with risk of high multicollinearity were excluded to avoid overfitting of the model, and the most parsimonious was selected.

*Step 3.* A cross-validation analysis was performed with multivariable CoxPH and backward conditional analysis selecting factors contributing to the primary model.

Finally, a set of 5 factors with significant and the most parsimonious univariate association including adjustment for treatment exposure were entered in the multivariable CoxPH (n = 127). All possible 2-way interactions between variables were explored (total of 10 possible interactions).

For internal validation of the model, 2 sensitivity analyses were performed. Both used a multivariable prognostic survival prediction machine learning model (ML-model) [19]. The first prognostic model discriminative ability was tested at 12 months as this coincided with the highest prevalence of patients that progressed. To analyze potential impact of sample size on the discriminative ability of different survival model algorithms, a second additional (boot-strapping) sensitivity analysis was performed by generating a 10-fold sample size increase based on randomly resampling 75% of events and censored non-events. For both sensitivity analyses, continuous and discrete time models were used of which two-thirds of the data was used to train the model (66.6/33.3 train/test data set). The various algorithms utilized were: neural network; Cox proportional hazards; random survival forest; gradient boosting machine; generalized linear model; conditional inference random forest; and support vector machine [19].

To assess the discriminative ability of the prognostic models, concordance index or area under the receiver operating curve (AUC) was used with 95% confidence interval (CI). These values can be interpreted as 0.5 no, > 0.5 poor, ≥ 0.7 acceptable, ≥ 0.8 excellent, ≥ 0.9 outstanding discrimination. The Brier score metrics will be used to assess overall performance considering calibration and discrimination of the final prediction model, lower values indicating better predictive performance [19]. The IBM SPSS Statistics for Windows (v29.0; UBM Corp, Armonk, NY, USA) were used for descriptive statistics of candidate prognostic variables at baseline. R Statistical Software (v4.2.2; R Core Team 2022; R Foundation for Statistical Computing, Vienna, Austria) in RStudio (v2023.09.1) was used for all primary analyses. R package survival v3.4–0 was used in the development of the prognostic CoxPH primary model and autoSurv v0.1.0 was used for the sensitivity analyses [19]. For univariate analysis statistical significance was considered if P < 0.1, while in all other tests statistical significance was considered if P < 0.05.

### Ethics, registration, data sharing, funding, use of AI, and disclosures

This study was conducted in accordance with the guidelines of the Declaration of Helsinki and approved by the Regional Ethical Board in Stockholm (Dnr 2012/172-31/4, 2015/1007-32, and 2017/609-32). Informed consent to participate was obtained from all participants included in the study. Trial registration ClinicalTrials.gov Identifier: NCT01761305 4 January 2013.

AA and PG were financially supported by the Swedish Research Council (Government funded competitive grant: Dnr 521-2012-1771); the regional agreement on medical training and clinical research (ALF) between Stockholm health care region & Karolinska Institutet (Government-funded competitive grants: FoUI-948087; FoUI-951336; FoUI-952892). PG: Swedish Society of Spinal Surgeons (non-profit NGO-funded competitive grant). AA: the Joanna Cocozza Foundation for Children’s Medical Research, Linköping University. The funders had no role in the design and conduct of the current study or approval and publication of the manuscript.

The de-identified dataset used and analyzed in the current study with definition of variables can be available on reasonable request after publication. Written proposals will be evaluated by the contributing authors. Approval of all authors will be required, and a data-sharing agreement must be signed beforehand. There was no use of generative artificial intelligence (AI) for the production of the current study.

Complete disclosure of interest forms according to ICMJE are available on the article page. doi: 10.2340/17453674.2024.41911

## Results

2,150 adolescents with idiopathic scoliosis were screened (Figure 2) and of 202 eligible patients, 67 declined participation leaving 135 for randomization. Of these 135 patients, 8 participants were censored due to loss to follow-up. In total 127 patients were analyzed whereof 14 had missing data in some baseline variables (Table 1–3).

### Prognostic outcome and follow-up time

The cohort consisted of 127 AIS patients with a Cobb angle of 25–40° (Figure 2) and total time to event (either skeletal maturity without curve progress or curve progression before skeletal maturity) for the cohort was a mean 19.5 months (SD 11.2). 76 patients (60%) did not progress vs 51 (40%) who progressed > 6° Cobb angle (Table 2), with a mean follow-up time of 23.6 (SD 10.7), and 13.5 months (SD 9.0), respectively. For each treatment group the event rate for curve progress was NB 11 (25%), SSE 19 (46%), and PA group 21 patients (50%). Tables 1–3 present descriptive statistics for the candidate prognostic variables collected at baseline, the criteria (step 1–3) for the prognostic model development of the primary model, and the covariate treatment exposure adjusted for in the final model.

### Risk factors in the primary multivariable prognostic model

The primary analysis displaying results for a multivariable CoxPH showed that the 4 factors related to the prognosis of curve progression were a Risser stage of 0 (HR 4.6, CI 2.1–10.1, P < 0.001), larger major curve Cobb angle (HR_standardized_ 1.5, CI 1.1–2.0, P = 0.005), and higher score on patient-reported pSAQ (HR_standardized_ 1.4, CI 1.0–1.9, P = 0.04), while premenarchal status contributed to the model but was not a statistically significant individual predictor (HR 2.1, CI 0.9–4.6, P = 0.08) (Table 4). Treatment exposure, entered as a covariate adjustment, contributed significantly (HR 3.1, CI 1.5–6.0, P = 0.001) (Table 4). Exploration of all possible interactions between variables showed no significant improvement of the model. The discriminative ability of the model between individuals with high or low risk of curve progression was defined as acceptable (concordance 0.79, CI 0.72–0.86).

**Table 4:** Primary prognostic multivariable Cox proportional hazards survival model (N = 127) with association between independent factors and curve progression (event, n = 51) adjusted for treatment exposure

Prognostic independent factors	HR (CI)	P value
Risser, Stage 0 vs 1–4 (Ref.)	4.6 (2.1–10.1)	< 0.001
Major curve Cobb angle (°)	1.5 (1.1–2.0) **^b^**	0.005
pSAQ (7–35)	1.4 (1.0–1.9) **^b^**	0.04
Menarche (boys coded as no),		
No vs Yes (Ref.)	2.1 (0.9–4.6)	0.08
Treatment exposure,		
SSE+PA vs NB (Ref.)	3.1 (1.5–6.0)	0.001
Concordance 0.79 (CI 0.72–0.86)		

For abbreviations, see Tables 1–3. Event: no progress if Cobb angle was ≤ 6° on reaching skeletal maturity or progress if curve progression > 6° occurred prior to skeletal maturity. Skeletal maturity was reached at < 1 cm of growth in body height over 6 months; CI: 95% confidence interval.

### Sensitivity analyses with machine-learning models

The CoxPH model maintained acceptable discriminative ability (AUC 0.79, CI 0.65–0.93) compared with the other machine learning algorithms with overall good predictive performance (Brier 0.15, CI 0.08–0.22) (Table 5). The bootstrapping sensitivity analysis with 10-fold simulated resampling showed slightly improved discriminative ability surpassing the cut-off for excellent level (AUC 0.84, CI 0.80–0.88), and predictive performance (Brier 0.14, CI 0.11–0.16) (Table 5). Several machine-learning algorithms improved towards an outstanding discriminative ability with increasing sample size (Table 5).

**Table 5:** Sensitivity analyses with multivariable prognostic survival prediction machine-learning models with association between independent factors and curve progression adjusted for treatment exposure, analyzed during a 12-month time-frame

	AUC (CI)	Brier (CI)
Sensitivity analysis 1, training data set n = 85; event n = 34 (40%)		
Gradient boosting machine	0.80 (0.66–0.93)	0.15 (0.09–0.21)
Cox proportional hazards	0.79 (0.65–0.93)	0.15 (0.08–0.22)
Generalized linear model	0.79 (0.65–0.93)	0.15 (0.09–0.22)
Neural network	0.77 (0.61–0.92)	0.16 (0.09–0.23)
Conditional inference random forest	0.75 (0.58–0.91)	0.16 (0.10–0.23)
Random survival forest	0.72 (0.53–0.90)	0.16 (0.10–0.23)
Support vector machine	0.69 (0.50–0.88)	0.17 (0.11–0.23)
Sensitivity analysis 2, training data set n = 634; event n = 252 (40%)		
Random survival forest	1.0 (0.99–1.00)	0.03 (0.02–0.04)
Gradient boosting machine	0.99 (0.98–1.00)	0.04 (0.03–0.05)
Neural network	0.97 (0.96–0.99)	0.06 (0.04–0.07)
Support vector machine	0.95 (0.92–0.97)	0.08 (0.06–0.10)
Conditional inference random forest	0.94 (0.91–0.97)	0.10 (0.08–0.11)
Cox proportional hazards	0.84 (0.80–0.89)	0.14 (0.11–0.16)
Generalized linear model	0.83 (0.79–0.88)	0.14 (0.12–0.16)

For abbreviations see Table 4. AUC: area under the receiver operating curve; Brier: assesses overall performance of the prediction models.

## Discussion

The aim of the present study was to develop an internally validated multivariable prognostic survival model exploring baseline variables for adolescent idiopathic scoliosis curve progression. The model created identified factors of importance for prognosing with acceptable discriminative and overall good predictive performance the risk of curve progression in AIS patients with a Cobb angle of 25–40°. After adjusting the model to treatment exposure, significant prognostic risk factors were Risser stage 0, larger major curve Cobb angle, and higher pSAQ score, while being premenarchal also contributed to the model but not significantly. These 4 prognostic factors were included from an initial 34 candidate variables spanning demographic, anthropometric, developmental, clinometric, radiologic, and patient-reported outcomes.

Apart from the prognostic value of Risser stages and curve magnitude previously reported in the literature [5,6,8], an interesting finding in the current study was that higher scores on the pSAQ predicted curve progress. The pictorial part of SAQ may capture other aspects of body image as it includes visual cues, which correlates better with the radiological severity compared with mere questions concerning appearance [20]. This might depend on the ability to integrate and perceive proprioceptive information and other sensory inputs such as vision to form one’s own body schema and to perceive body changes and appearance. Lonstein and Carlson [5] explored multivariable associations for 10° progress for mild scoliosis finding curve size, Risser, and age to be prognostic factors without analyzing discriminative ability. Our results showed pSAQ to be a stronger prognostic factor than age and variants thereof such as age-normalized growth variables, which were not retained in the final stage of the conditional multivariable survival analyses. Dolan et al. [4] applied logistic regression to untreated participants in the BrAIST study [3] showing Sander’s score, Cobb angle, and curve type to be strongly associated with curve progression (c-statistics 0.89–0.91). However, Risser sign was not included in the model development process and its prognostic value cannot be contrasted with the simplified skeletal maturity stage (SSMS) of Sanders et al. [21], or triradiate cartilage. Despite the excellent discriminative ability of Dolan and colleagues’ model [4] they did not test patient-reported measures or hereditary factors, and the major difference is that they defined progress as greater than 45° Cobb angle, which is a substantially more severe deformity than the progression of 6° cut-off in the current study. Parent et al. [22] retrospectively tested linear mixed-effect models on radiologic and demographic factors finding baseline Cobb angle, time, time2, age, curve type, and both interactions to predict larger future curves. An internal cross validation of Parent and colleagues’ model correctly predicted 80% of cases within 10° of curve progression to progress. The current study is able to predict a 6° progression with similar discriminative ability. The clinical implications of our model show the importance of adding patient-reported perceptions of spinal appearance to traditional clinical and radiological measurements to improve the discriminative ability of prognosticating curve progression toward a more patient-centered approach.

The current study is the only one to develop a full prognostic model based on time-dependent survival analysis for curve progression greater than 6° in a prospective cohort study on AIS patients with a Cobb angle of 25–40°. Associative factors do not necessarily point to causality in AIS, and it is difficult to determine whether associations are playing a role in the initial development of the AIS curve or are actual predictors of the progression [23]. The current study was designed as an exploratory prognostic cohort survival analysis within a completed multicenter RCT (CONTRAIS) that included high-quality data collected from baseline to endpoint. Accounting for treatment effect on the curve progression in the RCT [2], treatment was statistically adjusted for in our prognostic model. Our study population included ages 9–17, which reflects the normative pubertal growth phase as well as school screening routines in Sweden [24]. In our study participants had on average 2 years of skeletal growth left, which coincides with the peak growth phase where most risk of curve progression occurs. Both boys and girls were included in the study and had sex distribution representative of AIS prevalence. Considering that premenarcheal status contributed to the primary model, one may question the validity of this factor because 17% of the cohort were male and classified as pre-menarche. In terms of puberty, menarche in girls can be contrasted with spermarche in boys, described in the literature to occur at a median age of 14 years [25]. The mean age of boys in our cohort was 13.6 years and age-standardized time to < 1 cm height growth over 6 months was longer for boys than girls. This supports our assumption to code boys as pre-spermarche assuming the equivalence to pre-menarche in girls.

Due to the likelihood of AIS having a multifactorial etiology and pathogenesis, the current analysis included a broad range of prognostic variables. Our sample characteristics are to a large extent comparable to previous studies within the field [4,8,26] and for that reason our results may be applicable to AIS populations with similar inclusion criteria and healthcare systems. Our database covered various dimensions for prognostic analysis including novel factors capturing patient-reported measures on perception of trunk appearance, quality of life, and physical activity. Hence, our model included factors based on both existing knowledge [4,5,7] and candidate variables in order to improve the prognosis of future curve progression for the individual patient. The basic prerequisites for statistical power for our primary model were satisfied with adequate individuals per factor. Furthermore, univariate associations, acceptable normal distributions, and risk of multicollinearity were tested and found to be acceptable during the development process. After step-based preliminary analyses, the most associated factors were selected for the final model. The recommendations on transparent reporting of prognostic modeling were followed during the model development and internal validation process [10].

### Limitations

Additional radiographic parameters for SSMS [21] may have been a stronger predictor than Risser stages. But, on the other hand, additional radiographs could not be collected in the CONTRAIS study for ethical reasons because Risser was the standard measure when the study was planned in 2012. Despite the prospective nature of the dataset, the prognostic model could still be influenced by random noise and outliers. To mitigate this potential effect, the current analysis was internally validated by using ML-modeling [19,27]. Various algorithms and random resampling were utilized to assess the prognostic ability of our primary model. Algorithms in machine learning solve mathematical procedures using numerical computation, capturing the relationships between prognostic factors with the goal of predicting the event. These algorithms model nonlinear and complex relationships between prognostic factors as a method to minimize error. To apply ML models, large amounts of data need to be available to both learn and validate the actual model generated. While the current dataset was sufficient for our primary model, it might be insufficient to apply ML-model algorithms. A potential risk is overfitting the model, but the sensitivity analyses confirmed adequate discriminative ability with overall good predictive performance of the model. It is important to note that we measure data at 6-month intervals, which means a limited number of measurement points. Furthermore, external validation and research on the potential impact on patient outcomes are warranted. This remains to be tested in accordance with recommendations for prognostic model development before developing a risk calculator for broad clinical implementation [28].

### Conclusion

The prognostic model (Risser stage, Cobb angle, pSAQ, and menarche) predicted curve progression of > 6° with acceptable discriminative ability. Adding patient report of the pSAQ may be of clinical importance for the prognosis of curve progression.

### Supplementary data

Detailed description of interventions within CONTRAIS is available as Supplementary data on the article page. doi: 10.2340/17453674.2024.41911

## Supplementary Material



## Notes

Conceptualization: PG, AA, MD, DW, and HH; methodology: MD, AA, DW, and HH; software: AA; validation: AA, MD, and HH; formal analysis: MD and AA; investigation: ED, MD, AC, HT, AAA, HM, PG, and AA; resources: PG and AA; data curation: ED, MD, AC, HT, AAA, HM, PG, and AA; writing original draft preparation: MD and AA; writing review and editing: MD, HT, DW, HH, PG, and AA; visualization: MD and AA; supervision: BÖ, HT, PG, and AA; project administration: AA; funding acquisition: PG and AA. All authors have read and agreed to the published version of the manuscript.

The authors are grateful to all the patients for participating in this study. The authors give thanks to research coordinators Luigi Belcastro and Maria Wikzén; Ingrid Ekenman, Peter Endler, Panayiotis Savvides, Tomas Reigo, and Björn Dahlman for patient recruitments; and physiotherapists Sofia Berto and Suzanne Thorén.

Handling co-editors: Ilkka Helenius and Robin Christensen

*Acta* thanks James O Sanders, Stuart L Weinstein, and other anonymous reviewers for help with peer review of this manuscript.
